# Polytetrafluoroethylene and Aluminum Powder as an Alternative to Copper in Car Brake Composite Friction Materials—Part 2, Simulation Studies of Braking Process

**DOI:** 10.3390/ma19132756

**Published:** 2026-06-29

**Authors:** Andrzej Borawski

**Affiliations:** Faculty of Mechanical Engineering, Bialystok University of Technology, 45C Wiejska Str., 15-351 Bialystok, Poland; a.borawski@pb.edu.pl

**Keywords:** brakes, wear, friction, heat, temperature, simulation, theoretical studies

## Abstract

Currently, most design solutions are disc brake systems, in which, during braking, the rotating disc, along with the wheel, rubs against stationary brake pads, converting kinetic energy into thermal energy released into the atmosphere. Brake pads are made of composite materials. One of the key components is copper. Its presence is crucial and plays a crucial role in friction materials. In this work, an attempt was made to replace copper, which is unfortunately harmful to both the environment and humans, with aluminum powder and polytetrafluoroethylene powder. Samples of the proposed prototype friction materials were manufactured, and their thermal and tribological properties were determined (research described in the previous work). Knowledge of the materials’ properties allowed for simulation studies. Calculations were prepared for three different scenarios. The results showed that the heating process using the proposed materials during braking is very similar to that of materials with a conventional composition. Of the materials tested, composition where copper was replaced by polytetrafluoroethylene and aluminum in a 4:1 ratio gave the most promising results. In tests, this material had the lowest maximum brake pad temperature values, which contributes to a reduced risk of fading. Also, by “pushing” thermal energy into the brake disc, it contributes to the fastest dissipation of this energy. This suggests that the materials can be used in real-world braking systems.

## 1. Introduction

Brakes are extremely important devices. This applies primarily to vehicles, where their main task is to reduce speed, but also to bring the vehicle to a complete stop or maintain it stationary [1,2]. The correct operation and effectiveness of the braking system have a significant impact on road traffic safety [3,4]. Therefore, considerable attention is paid to friction materials and their properties.

Disc brakes are most often used in passenger cars, where brake pads mounted in a caliper rub against a disc rotating with the wheel [5]. The friction force generated depends on the brake fluid pressure in the system, which in turn depends on the driver’s pressure on the brake pedal. Brakes therefore act as a converter of mechanical and kinematic energy resulting from the vehicle’s motion into thermal energy, which is then dissipated to the environment [6,7]. To facilitate this, many manufacturers now use air ducts through which centrifugal force forces a flow, significantly improving convective heat transfer [8,9,10]. All tribological parameters related to the braking process are closely dependent on the friction pair materials. For the disc, gray cast iron is usually the choice. It is a good material, characterized by high mechanical properties, sufficient thermal conductivity, and is not expensive [11,12]. The brake pad material is a much more significant problem. Composite materials of various compositions and proportions are most commonly used [13,14]. Numerous studies show that a very wide range of components are used, from waste, e.g., from food production [15,16], through animal materials, e.g., hair or fur [17,18], combustion products, e.g., fly ash [19,20,21], to various types of metals [22,23] and ceramic components, e.g., silicon-based [24,25,26]. Generally, depending on the function performed in the composite friction material, components can be divided into several groups [27,28]:

- Reinforcement—a fibrous material primarily responsible for the mechanical strength of the final product;
- Matrix—most often resin, serves as the binder for the composite material;
- Fillers—components whose function is to fill the voids between other materials;
- And friction modifiers, which play a decisive role in determining the coefficient of friction.

One of the key friction modifiers currently used is copper. This material, thanks to its properties, is an almost mandatory component of brake pads. During friction braking, it is smeared across the disc surface, creating a thin lubricating film. This feature is important because it prevents accelerated wear, for example, due to adhesion. Furthermore, this lowers the coefficient of friction, thereby reducing the thermal load on the friction pair. Copper is also an excellent conductor. This feature also reduces thermal load, as heat is more quickly dissipated from the friction zone. Unfortunately, despite the above advantages, copper has a significant drawback: it is a harmful metal. Aquatic organisms are particularly sensitive to it, but it can also cause multi-organ diseases in humans. For this reason, the maximum copper content in brake pads is regularly reduced. The Euro 7 standard, scheduled for introduction in 2035, is intended to significantly reduce pollutant emissions from brake pads, in part by reducing copper content [29]. So-called non-exhaust emissions constitute a growing proportion of total emissions and now exceed exhaust emissions in many countries. Non-exhaust particulate emissions arise mainly from brake and tire abrasion, but also from road surface wear, and the re-dispersal of road dust [30]. This type of pollution may even result from the wear and tear of vehicle components, e.g., the engine [31,32].

Limiting the copper content in friction materials poses a serious problem and necessitates significant changes in brake pad design. Gilardi et al. used three types of graphite as a copper substitute. They used it as a solid lubricant in brake pads [33]. Six groups of samples were prepared—one without graphite, and one containing graphite in various forms and concentrations. Graphite proved to provide good lubrication properties. This helped, for example, reduce undesirable noise during braking. It also improved thermal conductivity. Mahale et al. took a slightly different approach. They decided to replace copper with stainless steel chips (SSS) [34]. They used their friction modifier proposal to develop a series of samples. Although the samples showed promising anti-adhesive properties, they unfortunately exhibited lower resistance to a key brake problem—fading. Different SSS concentrations also yielded different tribological properties—increasing the concentration resulted in an increase in the friction coefficient. Zheng et al. proposed a more ecological approach. A friction material was developed in which copper was replaced by using the properties of fly ash microspheres [35]. This significantly improved selected properties, such as thermal stability, hardness, and impact strength, reduced the density of the final product, effectively increased the coefficient of friction at medium and high temperatures, and improved the resistance of brake materials to fading. During the collaboration, the formation of a thin lubricating film on friction surfaces was also observed. Amit et al. used aluminum as a substitute for copper [36]. Four groups of samples were prepared, differing in the content of this element (0–15%). Both physical properties, such as mechanical strength and hardness, and tribological properties, were tested using a tribotester. A decrease in density and coefficient of friction was demonstrated with increasing aluminum concentration. Almost the same hardness, tensile strength, bending strength, and compressive strength were obtained, along with better stability in relation to the coefficient of friction. A similar idea was also developed by Strojny-Nędza et al. [37]. Four types of materials were developed with different percentages of aluminum and ceramic fraction. Their studies did not take into account tribological properties, focusing primarily on the porosity and hardness of the material.

Testing the working components of braking systems can be performed in many ways, using various tools. Simulation tests are most commonly used. They allow for the representation of very complex operating conditions, and such tests have a low overall cost. Computer simulations in the study of friction materials for brake pads enable in-depth analysis of mechanical and chemical processes at various scales—from the molecular to the macroscopic. These simulations enable prediction of material behavior under real-world conditions (often impossible or very difficult and costly), optimization of composition and structure, and resolution of problems related to issues such as durability and pollutant emissions. Modern models increasingly consider the impact of material degradation not only on tribological properties but also on dust generation and harmful emissions, which is crucial from the perspective of environmental regulations. Simulation studies increasingly combine molecular and microstructural simulations with macroscopic models of stress and wear analysis. This approach allows for understanding the relationships between the microstructural properties of a material and its behavior during real braking, enabling precise optimization of brake pad composition and structure. Current trends also focus on tribochemistry. Such models help predict how frictional properties change during operation and how to stabilize the coefficient of friction under varying conditions. It is also common practice to combine mechanical analyses with airflow simulations around and through the braking system, enabling better prediction of temperature distribution and cooling of friction elements. Such models support the design of systems that prevent overheating and material degradation.

Studies aimed at determining the frictional heating process are particularly popular. To reduce calculation errors, the method described by Stevens et al. [38], which uses first-order differential equations, can be used. You et al. found that different materials require different testing methods. They implemented their theory in studies of materials with different microstructures [39]. Esfe et al. demonstrated that, in addition to chemical composition, the frictional heating process is also influenced by the Reynolds number or particle size of the components used in production [40]. Similar studies were conducted by Kumar et al. [41]. They demonstrated a clear relationship between convective heat flow and the Reynolds number. It also appears that there is a relationship between the Nusselt number, the Reynolds number, and the disc-to-wheel diameter ratio, as investigated by Zhang et al. [42]. Ghandouri et al., in theoretical simulation studies, determined the effect of geometric features on the brake heating process. They used new types of ribs, which improved heat transfer coefficients [43]. Song et al. also introduced holes in the ribs, demonstrating a positive effect on the heat release rate [44].

Previous studies [45] have determined how replacing copper with polytetrafluoroethylene and aluminum powder affects the tribological properties of the proposed friction material. The literature contains studies on the use of aluminum in friction materials. Awe et al. [46] describe the use of aluminum in friction materials and its effect on the thermal conductivity and wear resistance of brake materials. The results indicate that aluminum improves heat dissipation, which reduces the risk of overheating and pad cracking, while stabilizing the friction coefficient. Pacino et al. [47] also demonstrated that the addition of aluminum allows for a reduction in component weight while maintaining good durability and braking efficiency, which is particularly attractive in electric vehicles. The aim of this study is to continue this research by determining the friction heating process of the proposed composite containing both aluminum and polytetrafluoroethylene. A virtual environment will be used for this purpose, and the necessary input data will be determined experimentally using material samples.

## 2. Materials and Methods

The research described in this article is purely theoretical. The simulation input parameters were based on laboratory tests of real composite friction materials. The object of the study was an actual passenger car braking system.

The idea is to reduce, or ideally eliminate, copper (which in our study consisted of a powder with a purity of >99% and a grain size of <60 µm) from the composition of this material. Polytetrafluoroethylene powder (CF2CF2Jn, grain size 20–70 µm) and aluminum powder (Al, grain size ~65 µm) were chosen.

Aluminum was chosen for its excellent thermal conductivity and density. Low density allows for weight reduction, which is particularly important in vehicles, as it directly affects inertia and thus contributes to both improved acceleration and deceleration. Aluminum also has a low Young’s modulus (approximately 40% compared to copper), making it more malleable. This facilitates the shaping of the final product. Aluminum’s anti-corrosion properties are also important, positively impacting the service life of the braking system.

Polytetrafluoroethylene (Teflon) also possesses many desirable properties in braking systems. Primarily, it has excellent lubricating properties. This property contributes to a reduction in the coefficient of friction, which in turn positively impacts the thermal load on the brake components. Another important characteristic is its resistance to the vast majority of known solvents. Teflon is unaffected by changes in UV radiation intensity and is also resistant to high temperatures, remaining unchanged up to 250–300 °C. It is also environmentally friendly, being odorless, tasteless, and non-toxic. In addition to the above key components, the following materials were used in the study: ground steel with a grain size of 0.5–1.0 mm, aramid fibers (thickness <0.1 mm, length ~5 mm), polyester resin, fly ash (grain 1–200 µm), and gray cast iron powder (EN-GJS-400-12, grain <200 µm). All materials were manufactured by SYNTHETIKA SP.Z.O.O, Łódź, Poland.

Four types of samples (S1–S4) were prepared for the study. The main differences between the groups are as follows:

- S1 samples had a fairly conventional composition, with 20% copper as the lubricant and heat-dissipating component;
- In the S2 samples, copper was eliminated, and its properties were replaced by aluminum powder for thermal conductivity and Teflon powder for lubrication;
- In the S3 group, copper was also eliminated, but the aluminum powder content was increased at the expense of polytetrafluoroethylene powder;
- S4 samples had the highest aluminum content and the lowest tetrafluoroethylene content of all the samples tested; copper was also omitted in this case.

The proportions (Table 1) were measured using a Steinberg SBS-LW-300A laboratory scale (accuracy 10^−3^ g, Steinberg, Hamburg, Germany). Dedicated containers were used for weighing, which were washed and dried before each measurement to improve measurement precision. Techniplast 400 epoxy resin was used to prepare the samples (product data sheet available on the manufacturer’s website).

**Figure 1:**
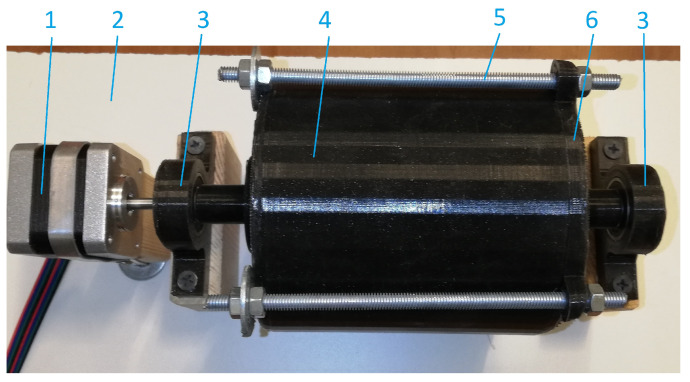
3d printed mixing device for composite raw materials: 1—stepper motor, 2—base, 3—bearing, 4—mixing tube, 5—connecting screw, and 6—lid.

To ensure the highest possible homogeneity of the sample material, an additive mixing device was used (Figure 1). The material was left in the mixing chamber for one hour. A stepper motor set the speed to 50 RPM (by external Arduino driver), which, combined with internal ribs, allowed for thorough mixing and thus even distribution of the individual components.

The prepared material was placed in molds. A hydraulic press was then used to apply 20 MPa of pressure to the material. After full curing, the samples were demolded and heated at 55–60 °C for 24 h. The purpose of this was to evaporate residual chemicals, such as solvents used for cleaning containers, and to finally cure the resin. Two versions of the samples were prepared:

- Cylindrical with a diameter of 1″ (25.4 mm) and a height of 10 mm, which were used for tribological testing;
- And cuboidal with a side length of 20 mm, used for testing thermal and physical properties.

**Figure 2:**
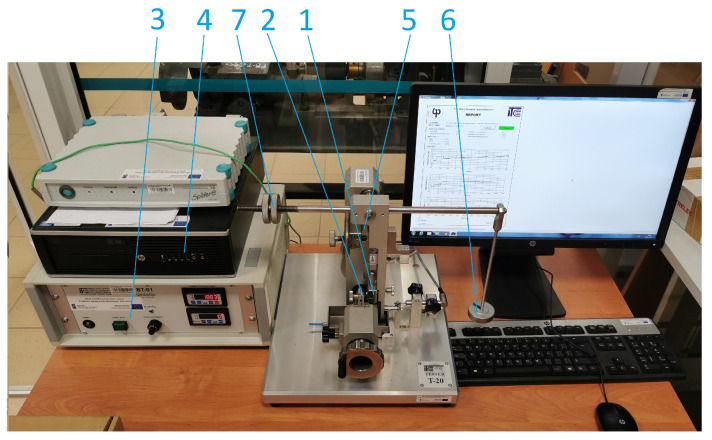
T-20 test stand used in experiment: 1—sample, 2—counter sample, 3—control unit, 4—PC, 5—motor, 6—load, 7—lever with counterweight.

The first stage of the research, aimed primarily at determining the coefficients of friction for individual sample groups, was performed using a T-20 laboratory stand (ball-disc friction pair, Figure 2).

The experimental Taguchi multiparameter process optimization method was used to develop the research plan. This method assumes the adoption of an orthogonal table based on a number of variables (in this case, three: sample contact force, friction path, and friction velocity), and based on this table, preliminary tests are conducted. These tests allow for the determination of input parameter values for the main experiment, which provides the best repeatability of results, i.e., the smallest relative error. After selecting the optimal input parameters, the main experiment was conducted, in which multiple repetitions allowed for the determination of mean friction coefficient values along with standard deviation values. The detailed research procedure is described in a previous publication [45]. The obtained results are summarized in Table 2.

Each simulation study requires input parameters. Their number may vary depending on the complexity of the mathematical model, but the essential parameters that directly influence the friction heating process include density, thermal conductivity, and thermal capacity [48,49]. These parameters were determined by an external company, and the results are presented in Table 3.

To determine the course of the friction heating process, it was necessary to develop a mathematical model. Vehicle speed is closely dependent on the rotational speed of the wheels, while the torque required to ensure adequate braking can be expressed as:

(1) $$M_{f}\left(t\right)=-I\frac{d\omega \left(t\right)}{dt},\;t_{1}\;\leq t\;\leq \;t_{2}$$

where *ωt* is the wheel rotational speed, *I* is the torque of inertia taking into account the energy *E_k_*(*t*). The moment of inertia was calculated after the transformation:

(2) $$I=\frac{2E_{k}(t)}{\omega t^{2}r_{c}},\;t_{1}\;\leq t\;\leq \;t_{2}$$

where *r_c_* is the average radius of engagement of the disc and brake pad. It is determined by the relationship:

(3) $$r_{c}=\frac{2\pi }{A}r^{2}dr=\frac{2(r_{2}^{3}-r_{1}^{3})}{3(r_{2}^{2}-r_{1}^{2})},\;r_{1}\;\leq r\;\leq \;r_{2}$$

where *r*_1_ is the minimum contact radius of the disc and pad, and *r*_2_ is the maximum. The instantaneous kinetic energy of the vehicle moving at speed *v* is determined as follows:

(4) $$E_{k}\left(t\right)=\frac{mv^{2}}{2},\;t_{1}\;\leq t\;\leq \;t_{2}$$

The braking torque can also be written using the formula:

(5) $$M_{f}(t)=fpAr_{c},\;t_{1}\leq t\leq t_{2},$$

where *f*—coefficient of friction between the disc and the pad determined experimentally, *p*—contact pressure, *A*—contact surface area of the disc and the pad. By transformation, the contact pressure can be determined:

(6) $$p(t)=\frac{M_{f}(t)}{Ar_{c}},\;t_{1}\;\leq t\;\leq \;t_{2}$$

The contact surface area is determined by the formula:

(7) $$A=2\left(\frac{\propto_{1}}{\pi }\left(r_{2}-r_{1}\right)\right)(r_{2}-r_{1})$$

where *α*_1_ is the contact angle of the pad on the friction surface. The friction power, in turn, could be determined from the relationship:

(8) $$P(t)=fpAV(t),\;t_{1}\leq t\leq t_{2},$$

where *V* is the linear velocity of the friction pair at the central point of contact. This velocity can also be written as follows:

(9) $$V(t)=\omega (t)r_{c},\;t_{1}\leq t\leq t_{2},$$

Finally, taking into account the above equations, the power can be determined by the equation:

(10) $$P\left(t\right)=fp\omega \left(t\right)r_{c},\;t_{1}\leq t\leq t_{2},$$

During braking, heat is generated at the expense of reducing the vehicle’s kinetic energy. At time *t*_2_, i.e., when the vehicle comes to a complete stop, the loss of kinetic energy is equal to the work done by the braking system, i.e.,

(11) $$E_{k}\left(t\right)=-∆U,\;t_{2}\leq t,$$

where *ΔU* is the work done by the braking system to bring the vehicle to a complete stop. In the case under study, the following equation was developed using the Fourier–Kiechhoff law to determine the heat distribution in the friction materials:

(12) $$\frac{\partial^{2}T}{\partial r^{2}}+\frac{1}{r}\frac{\partial T}{\partial r}+\frac{1}{r^{2}}\frac{\partial^{2}T}{\partial \propto^{2}}+\frac{\partial^{2}T}{\partial z^{2}}=\frac{1}{k_{d}}\frac{\partial T}{\partial t}+\omega \frac{\partial T}{\partial \propto },\;t_{1}\leq t\leq t_{2},\;r_{1}\leq r\leq r_{2},\;\alpha_{0}\leq \alpha \leq \alpha_{1},\;z_{0}\leq z\leq z_{1}.$$

The following initial and boundary conditions were assumed in the considerations:

(13) $${\left.\frac{\partial T}{\partial z}\right|}_{z=z_{0}}=0,\;t_{1}\leq t\leq t_{2},\;r_{1}\leq r\leq r_{2},\;\alpha_{0}\leq \alpha \leq \alpha_{1},z=z_{0}$$

(14) $${\left.K_{d}\frac{\partial T}{\partial r}\right|}_{r=r_{1}}=h[T\left(z,\alpha ,t\right)-T_{a}],\;t_{1}\leq t\leq t_{2},\;z_{0}\leq z\leq z_{1},\;\alpha_{0}\leq \alpha \leq \alpha_{1}$$

(15) $${\left.K_{d}\frac{\partial T}{\partial r}\right|}_{r=r_{2}}=h[T_{a}-T\left(z,\alpha ,t\right)],\;t_{1}\leq t\leq t_{2},\;z_{0}\leq z\leq z_{1},\;\alpha_{0}\leq \alpha \leq \alpha_{1}$$

(16) $${\left.K_{d}\frac{\partial T}{\partial z}\right|}_{z=z_{0}}=q_{d}\left(t,r,\alpha \right),\;t_{1}\leq t\leq t_{2},\;r_{1}\leq r\leq r_{2},\;\alpha_{0}\leq \alpha \leq \alpha_{1}$$

(17) $${\left.K_{d}\frac{\partial T}{\partial z}\right|}_{z=z_{1}}=h[T_{a}-T\left(r,\alpha ,t\right)],\;t_{1}\leq t\leq t_{2},\;r_{1}\leq r\leq r_{2},\;\alpha_{0}\leq \alpha \leq \alpha_{1}$$

where *T*—temperature, *T_a_*—ambient temperature, *h*—thermal conductivity coefficient, *K_d_*—thermal conductivity of the disc, and *q_d_*—heat flow intensity in the disc, which was determined as follows:

(18) $$q_{d}=\gamma f\left(T\right)p\left(t\right)V\left(t\right),$$

where *γ* is defined by the proportionality factor. Taking into account the fact that the disc performs a rotational motion, the linear friction velocity can be written as the product of the rotational velocity and the mean radius of engagement, obtaining:

(19) $$q_{d}=\gamma f\left(T\right)p\left(t\right)\omega \left(t\right)r_{c}.$$

The proportionality coefficient of heat distribution in the friction pair is described by the Charron’s equation which takes the following form [50,51,52]:

(20) $$\gamma =\frac{\sqrt{K_{d}\rho_{d}c_{d}}}{\sqrt{K_{d}\rho_{d}c_{d}}+\sqrt{K_{p}\rho_{p}c_{p}}}$$

where *K_p_*—thermal conductivity of the pad, *K_d_*—thermal conductivity of the disc, *ρ_p_*—density of the pad material, *ρ_d_*—density of the disc material, *C_p_* and *C_d_*—heat capacity of the pad and the disc.

The tests performed and described in this paper are comparative in nature. The same initial braking conditions were assumed for all tested samples (S1–S4), i.e., three cases:

(a) emergency braking in urban traffic from an initial speed of 50 km/h,
(b) emergency braking in intercity traffic from an initial speed of 90 km/h,
(c) emergency braking on a highway from an initial speed of 140 km/h.

Due to the limitations of the adopted test method, it was necessary to adopt a number of simplifying assumptions. These included:

- Constant contact pressure of the friction pair;
- The pad surface is perfectly flat and friction occurs across the entire surface of the brake pad;
- A constant ambient/initial temperature of 293.15 K (20 °C),
- Braking occurs without wheel slippage, and the braking deceleration is constant and equal to the acceleration of gravity (dry road, the coefficient of friction between the tire and the road is 1);
- The disc and pad materials are homogeneous;
- Braking occurs without the influence of external factors such as road irregularities, gusts of wind, or hills;
- Thermal contact was assumed to be perfect—all friction energy was converted into heat;
- The coefficient of friction of the working brake elements is not temperature-sensitive and is constant throughout the braking process; in real conditions, the friction coefficient changes significantly and can drop to almost zero, but these studies are of a comparative nature, so the above simplification was considered justified.

**Figure 3:**
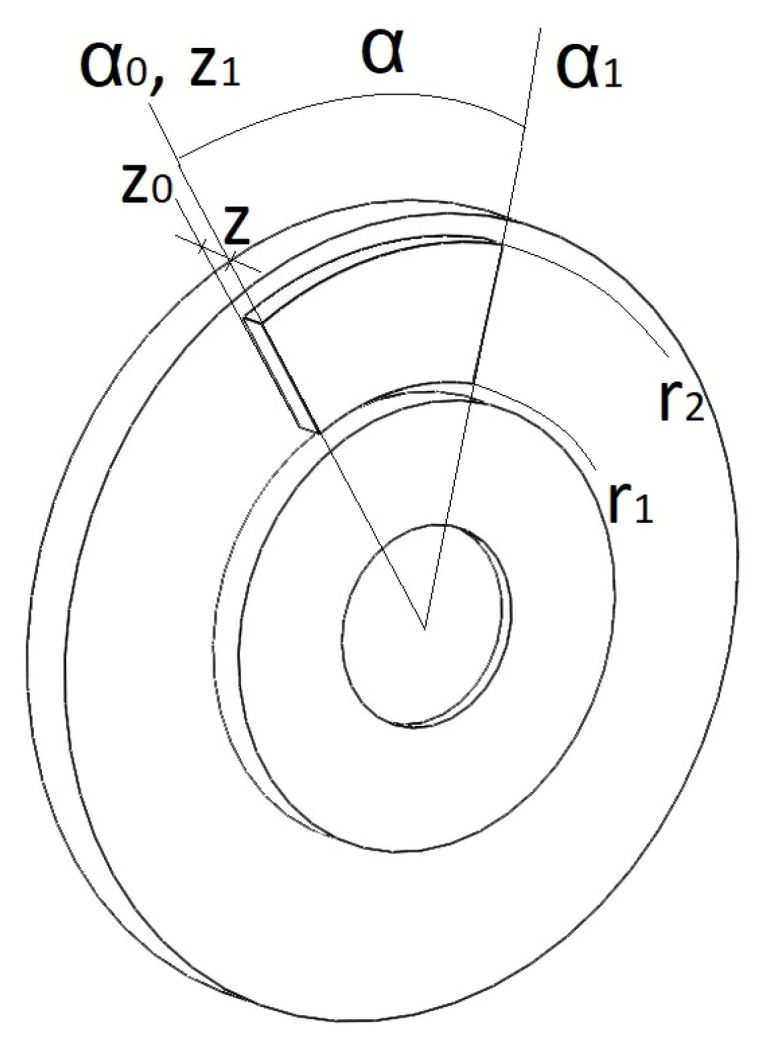
3d model of brake disc and pad.

The geometry of the research model, i.e., the dimensions of the brake disc and brake pad, was based on a popular European passenger car. The vehicle’s curb weight was also assumed in the calculations. The 3D model was developed in the SolidWorks 2025 environment and then implemented in Comsol Multiphysics 5.6 software (Figure 3).

To reduce testing time, the model used in the study was slightly modified compared to the original parts: roundings, chamfers, and mounting holes were removed. Additionally, the brake pad guides that position it in the brake caliper were removed. Due to their characteristics and intended use, these changes allowed for a balance between the quality of results and the effectiveness of the tests. This prepared test object allows for a focus on frictional heating and the factors that have the greatest impact on the results, and also enables faster testing of various material variants and operating conditions.

The characteristic geometric quantities used in the calculations are summarized in Table 4.

## 3. Results and Discussion

**Figure 4:**
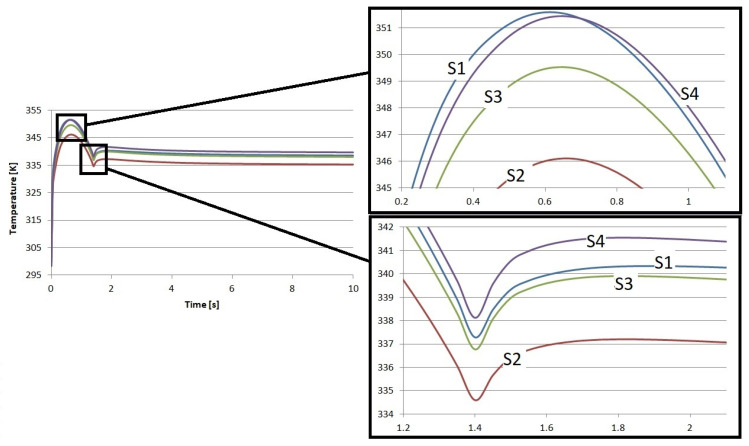
FEM calculation results of brake pad friction heating—initial speed 50 km/h.

**Figure 5:**
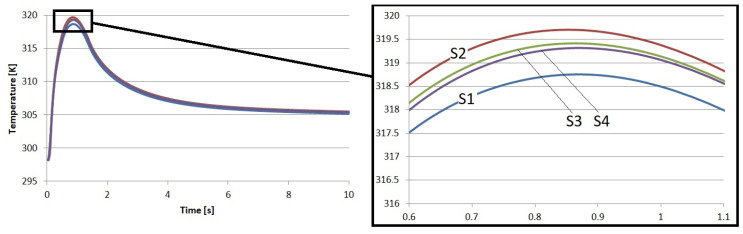
FEM calculation results of brake disc friction heating—initial speed 50 km/h.

A series of numerical studies using the finite element method was performed. The simulations were based on a developed mathematical model of the process and the adopted simplifying assumptions. The studies were performed on four groups of prototype samples, each in three different scenarios. The 3D test object, prepared in the COMSOL 5.6 environment, with an applied mesh, had nearly 30,000 degrees of freedom (DoF’s). In each case, the first 10 s from the start of braking were simulated. Temperature measurements were taken at the geometric center of the interacting surfaces, 0.05 mm below the surface in both materials. Figure 4 and Figure 5 present the results of numerical calculations of frictional heating during braking from an initial speed of 50 km/h.

**Figure 6:**
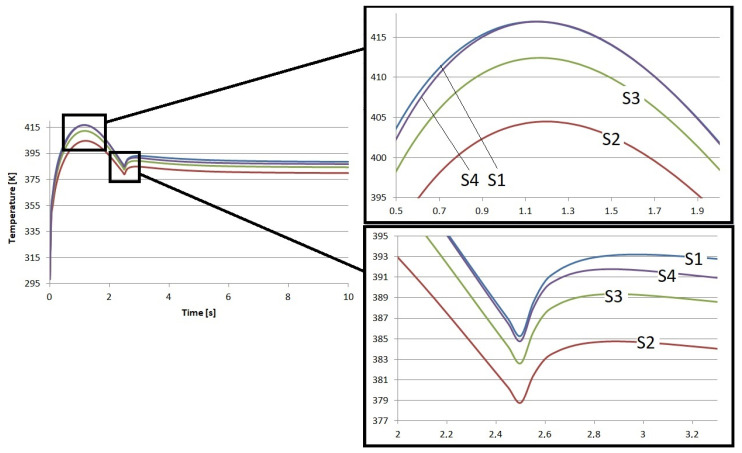
FEM calculation results of brake pad friction heating—initial speed 90 km/h.

**Figure 7:**
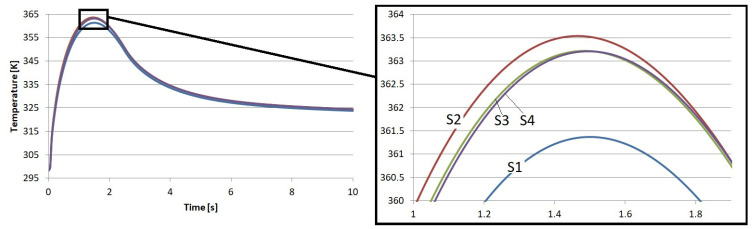
FEM calculation results of brake disc friction heating—initial speed 90 km/h.

The highest brake pad temperature observed was for sample S1, just over 351.5 K. This was reached after only 0.6 s from the start of braking. Interestingly, for the same sample, the disc temperature was the lowest of all measured, reaching just under 319 K. The coolest brake pad was found with sample S2 material. The difference compared to S1 was only about 1 K, so it can be assumed that it will not significantly impact the driver’s experience. A similar difference was noted for the brake disc. Importantly, it is clear that sample S2 material better transfers thermal energy to the disc—the brake pad temperature at the moment of complete stopping (change in the graph direction) was the lowest. Figure 6 and Figure 7 present the calculation results for an initial speed of 90 km/h, i.e., in rural conditions.

At a higher initial speed, the vehicle had significantly more kinetic energy, which the braking system had to dissipate [53]. Therefore, the final temperatures, particularly of the brake pad, were higher. In this case, sample S1 also heated up the most. Interestingly, sample S4 reached the same temperature. They differed only in the slope of the heating curve—sample S1 reached its maximum temperature slightly faster. Later in the testing process, the temperature curves for S1 and S4 almost overlap. The difference becomes visible after braking, where the S4 material releases heat more quickly. However, significant differences are visible in the brake disc. When working with S1, the disc heated to 361.37 K, while with S4, it reached 363.21 K. This difference is insignificant (approximately 1%), but noticeable in the graphs.

**Figure 8:**
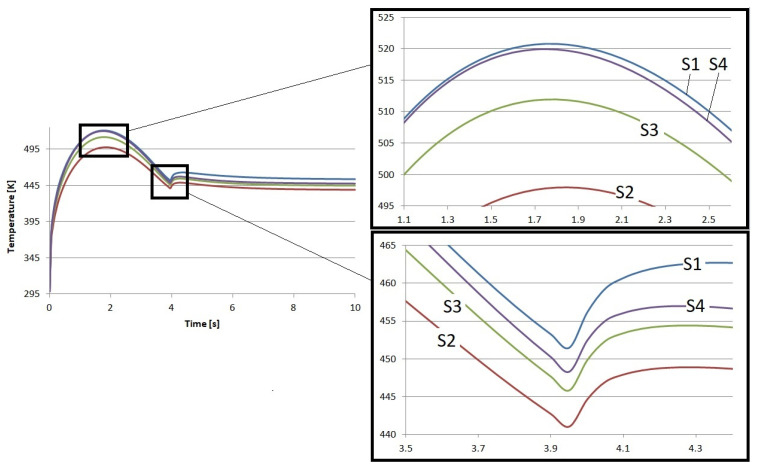
FEM calculation results of brake pad friction heating—initial speed 140 km/h.

**Figure 9:**
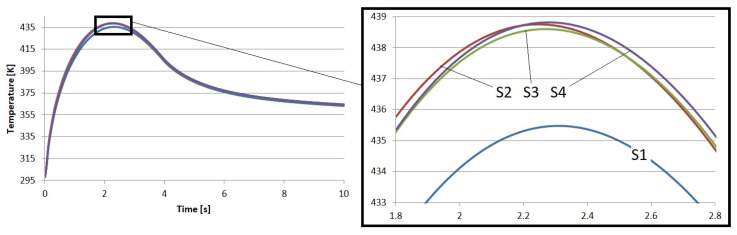
FEM calculation results of brake disc friction heating—initial speed 140 km/h.

As with the lower speed, the brake pad made from sample S2 also showed the lowest maximum temperature, reaching 404.49 K. The temperature profile of the disc clearly shows that the thermal energy was absorbed by the disc, which in this test heated to a temperature of 363.53 K. The results of further increasing the speed to the motorway speed limit of 140 km/h are presented in Figure 8 and Figure 9.

In the latter case, the pad made of sample S1 also heated up the most, reaching as much as 520.73 K. Unfortunately, this is a very high temperature and in many cases carries a serious risk of fading. The pad made of material S4 heated up slightly slower and, as a result, less intensely. Its maximum temperature was 519.9 K. As in previous studies, the pads made of material S2 also achieved the lowest temperature, reaching 497.92 K. Regarding the brake disc, the temperature curves for materials S2–S4 were almost identical. Only minor deviations were observed, which had no significant impact on the vehicle’s braking process. However, a clearly noticeable deviation in the temperature profile of the brake disc when working with material S1 was slightly over 3 K.

It’s worth noting that the friction process between brake pads and discs involves complex mechanical, thermal, and chemical phenomena. The high temperature generated at the contact point causes changes in the microstructure of the materials and can trigger tribochemical reactions, leading to the formation of thin layers on the friction surfaces. Furthermore, during braking, microscopic material adhesion and detachment occur (so-called adhesion and abrasion phenomena), generating microdust and affecting the service life of the components. Consequently, the friction process is a dynamic interaction of contact mechanics, heat transfer, and chemical reactions, which determine the effectiveness and durability of the braking system. The theoretical research conducted by the author does not take these facts into account.

## 4. Conclusions

This article presents the results of temperature simulation tests on a friction pair consisting of a brake disc and pad. Actual prototype materials were used as the pad materials to determine their tribotechnical properties. These materials are an innovative attempt to replace copper with polytetrafluoroethylene and aluminum powder. FEM calculations were performed for three scenarios with different initial speeds. The study determined that:

1. All materials exhibit promising properties, suggesting they could be successfully used in brake pad production.
2. Polytetrafluoroethylene and aluminum, under current thermal simulation and partial experimental conditions, may become a candidate solution as a copper replacement in friction materials. Their use has no significant impact on the heating process of the friction pair.
3. Of the materials tested, material S2 performed best, where copper was replaced by polytetrafluoroethylene and aluminum in a 4:1 ratio. This resulted in the lowest maximum brake pad temperature values, which contribute to a reduced risk of fading.
4. The S2 material, by “pushing” thermal energy into the brake disc, contributes to the fastest dissipation of this energy. Mechanical testing of the proposed materials is planned for the next stage, which will provide information on their resistance to loads occurring in vehicle braking systems.

## Figures and Tables

**Table 1:** Compositions of samples S1–S4.

Component	S1 (wt.%)	S2 (wt.%)	S3 (wt.%)	S4 (wt.%)
Copper	20	0	0	0
Aluminum	0	4	10	16
Polytetrafluoroethylene	0	16	10	4
Steel (0.18% C, 0.5% Si, 1.65% Mn, 0.05% P, 0.02% S, 0.08% Mo)	11	11	11	11
Aramid fiber	18	18	18	18
Resin	19	19	19	19
Fly ash, grain 1–200 µm	23	23	23	23
Cast iron powder	9	9	9	9

**Table 2:** Average coefficient of friction values.

Group No	Average COF	Standard Deviation
S1	0.384	±0.0124
S2	0.237	±0.0324
S3	0.312	±0.0157
S4	0.353	±0.0144

**Table 3:** Physical and thermal data of friction materials.

Parameter	S1	S2	S3	S4	Disk
Thermal conductivity [W/m·K]	119	123	126	129	45
Density [kg/m^3^]	2970	2935	2905	2880	7850
Heat capacity at constant pressure [J/kg·K]	1248	1176	1141	1110	460

**Table 4:** Geometrical data used for calculations.

Parameter	Value
Minimum friction radius, *r*_1_	235 mm
Maximum friction radius, *r*_2_	150 mm
Brake pad thickness, *z*	10 mm
The angle between the edges of the brake pad, *α*	45 deg
Vehicle mass, *m*	1200 kg

## Data Availability

The original contributions presented in the study are included in the article; further inquiries can be directed to the author.
